# Molecular Mechanisms of Liver Metastasis: Connecting Biology, Biomarkers, and Outcomes

**DOI:** 10.3390/cells15050469

**Published:** 2026-03-05

**Authors:** Doris Wagner, Felix Balzer, Georgios Antonios Margonis

**Affiliations:** 1 Department of General Surgery, Medical University of Graz, 8036 Graz, Austria; doris.wagner@medunigraz.at; 2 Institute of Medical Informatics, Charité—Universitätsmedizin Berlin, Corporate Member of Freie Universität Berlin and Humboldt-Universität zu Berlin, 10115 Berlin, Germany; felix.balzer@charite.de; 3 Sloan School Operations Research Center, Massachusetts Institute of Technology, Boston, MA 02142, USA; 4 Department of Surgery, Memorial Sloan Kettering Cancer Center, New York, NY 10065, USA

In our Special Issue titled “Molecular Mechanisms of Liver Metastases,” we aimed to attract articles that connect metastasis mechanisms and biomarkers with clinical disease characteristics and patient outcomes. Starting with the review paper by the Ohio State group, the authors provide valuable insight into the complex, multi-step molecular mechanisms underpinning liver metastasis [1]. Moving from pathogenesis to the prognostic and predictive role of clinically actionable proxies of tumor biology, the University of California, San Francisco (UCSF) group reviews current evidence on the prognostic and predictive relevance of key alterations—including *RAS*, *BRAF*, mismatch repair (MMR) genes, *TP53,* and *SMAD4*—in surgically treated colorectal liver metastases (CRLMs) [2]. For *RAS* and *BRAF* in particular—given the breadth of existing evidence—the authors emphasize the largest and/or the most recently published studies to provide the most robust and contemporary overview for readers.

A central message emerging from this work is two-fold. First, heterogeneity within *KRAS* and *BRAF* matters: exon-, codon-, and nucleotide-specific variants have been associated with distinct prognostic implications, underscoring that these biomarkers cannot be treated as uniform entities. Second, the prognostic and predictive role of somatic alterations is likely context-dependent, shaped by multidimensional relationships among co-occurring mutations and other biological factors. Some alterations may not be informative in isolation, and their clinical meaning may only become apparent when interpreted within a broader molecular and clinical context—one that may be too complex to capture using traditional, low-dimensional statistical approaches.

The subsequent contribution by Underwood and colleagues shifts attention toward therapeutic implications: as additional mutations and oncogenic proteins are discovered in metastatic colorectal cancer (mCRC), new targets for precision therapies will continue to emerge [3]. Importantly, this perspective complements the UCSF review by emphasizing that the clinical relevance of tumor biology extends beyond risk stratification to the identification of actionable vulnerabilities—and that the set of actionable targets in mCRC is likely to expand as molecular understanding deepens.

While the UCSF paper focuses primarily on tissue-based proxies of tumor biology in CRLM, the meta-analysis from the Rotterdam group takes this concept a step further by focusing not on static tissue biomarkers, but on dynamic biomarkers that can be measured longitudinally across a patient’s clinical trajectory [4]. Specifically, they synthesize evidence on circulating tumor DNA (ctDNA) for recurrence prediction before surgery, after surgery, and following adjuvant systemic therapy. Their findings support a clear association between detectable ctDNA after surgery and/or after adjuvant chemotherapy and worse outcomes—higher recurrence risk and shorter overall survival—whereas the prognostic implications of preoperative ctDNA remain less consistent across studies. Importantly, the authors highlight a point that is not always fully acknowledged in clinical discussions: recurrence rates among patients with undetectable ctDNA after treatment are not negligible, suggesting that, at present, adjuvant chemotherapy should not be omitted based on negative postoperative ctDNA alone.

Taken together, these papers converge on a fundamental challenge in CRLM—and, more broadly, in oncology. The biomarkers we currently measure likely capture only a fragment of the true biological landscape driving metastasis, treatment response, and recurrence. Many relevant factors remain unmeasured, and we often do not know how unobserved factors interact with the biomarkers we do assess. This gap plausibly contributes to the variability observed across studies. For example, specific *KRAS* variants (e.g., G12V) have been reported as aggressive in some surgical CRLM cohorts, yet appear less consistently prognostic in randomized trial settings [5,6]. The MD Anderson group publications [7,8] further suggest that the impact of *KRAS* may depend on co-alterations such as *TP53*—while it is equally plausible that the true interaction structure is even more complex than what we can currently observe and model.

This Special Issue therefore serves two purposes: it consolidates the current evidence base and reports the heterogeneity and uncertainty that persist—while also underscoring the need to resolve these issues, both to deepen our biological understanding and to improve patient outcomes. How can the field move forward? In our view, two complementary approaches are particularly promising.

First, we must expand molecular characterization—ideally through broad next-generation sequencing (NGS)—to more comprehensively map the key drivers of CRLM biology. However, this will take time: comprehensive profiling is not uniformly implemented across centers, and even when molecular data are available at scale, extracting reliable interaction signals is analytically challenging. High-dimensional settings with many candidate molecular variables and comparatively limited patient numbers are vulnerable to the “curse of dimensionality,” and global feature-importance approaches can obscure clinically meaningful effects that exist only within specific subgroups. Yet identifying such subgroups—patients for whom a biomarker truly matters—is precisely the essence of precision medicine.

Second, and in parallel, we believe advanced methodological approaches can help address the “context” problem created by unmeasured factors. By “advanced methods,” we do not mean machine learning and AI per se: when naively applied to real-world data—the most commonly used data source in clinical research due to its abundance—these models do not resolve the biases inherent in such data, but may instead propagate them. What we mean is optimization-based and causal-inference frameworks that can leverage real-world data to emulate key properties of randomized trials by balancing prognostic factors across treatment groups—even when some of those factors are not directly measured. Conceptually, this is what trials do: randomization does not require measuring every prognostic determinant; rather, it balances both observed and unobserved prognostic factors across arms, allowing valid causal conclusions about the treatment under study. Modern debiasing approaches aim to approximate that balance in observational cohorts. This, in turn, can help clarify not only the true treatment effect of an intervention—as RCTs are designed to do—but also the “core” prognostic or predictive value of specific variants (e.g., *KRAS* subtypes) by reducing distortion from confounding biological context.

A natural next step after such debiasing is to move from average effects to actionable stratification: interpretable AI models—such as decision-tree-based approaches—may identify combinations of available clinical and molecular features that act as practical surrogates for otherwise unobserved biology [9]. For instance, a *KRAS* variant combined with tumor burden metrics and patient characteristics might delineate a subgroup likely to harbor additional, unmeasured alterations—and thereby approximate the biological context that we cannot directly observe. We are actively pursuing this line of work and hope to report these advances in the near-future.
